# Supplementary material for: Insights into the molecular triggers of parosmia based on gas chromatography olfactometry
Source: Commun Med (Lond). 2022 May 24;2:58. doi: 10.1038/s43856-022-00112-9 (PMC9130211; doi:10.1038/s43856-022-00112-9)
Supplement: Supplementary file 1 — Reporting Summary [file 43856_2022_112_MOESM1_ESM.pdf]

nature research

Corresponding author(s): *Double-blind peer review submissions: write DBPR and your manuscript number here instead of author names.*

Last updated by author(s): *YYYY-MM-DD*

Reporting Summary

Nature Research wishes to improve the reproducibility of the work that we publish. This form provides structure for consistency and transparency in reporting. For further information on Nature Research policies, see our Editorial Policies and the Editorial Policy Checklist.

Statistics

For all statistical analyses, confirm that the following items are present in the figure legend, table legend, main text, or Methods section.

n/a	Confirmed	
☐	☒	The exact sample size (*n*) for each experimental group/condition, given as a discrete number and unit of measurement
☐	☒	A statement on whether measurements were taken from distinct samples or whether the same sample was measured repeatedly
☒	☐	The statistical test(s) used AND whether they are one- or two-sided<br>*Only common tests should be described solely by name; describe more complex techniques in the Methods section.*
☒	☐	A description of all covariates tested
☐	☒	A description of any assumptions or corrections, such as tests of normality and adjustment for multiple comparisons
☐	☒	A full description of the statistical parameters including central tendency (e.g. means) or other basic estimates (e.g. regression coefficient) AND variation (e.g. standard deviation) or associated estimates of uncertainty (e.g. confidence intervals)
☒	☐	For null hypothesis testing, the test statistic (e.g. *F*, *t*, *r*) with confidence intervals, effect sizes, degrees of freedom and *P* value noted<br>*Give P values as exact values whenever suitable.*
☒	☐	For Bayesian analysis, information on the choice of priors and Markov chain Monte Carlo settings
☒	☐	For hierarchical and complex designs, identification of the appropriate level for tests and full reporting of outcomes
☒	☐	Estimates of effect sizes (e.g. Cohen's *d*, Pearson's *r*), indicating how they were calculated

Our web collection on statistics for biologists contains articles on many of the points above.

Software and code

Policy information about availability of computer code

Data collection	No computer code was generated.
Data analysis	All statistics were carried out using XLStat v 20201.1.1

For manuscripts utilizing custom algorithms or software that are central to the research but not yet described in published literature, software must be made available to editors and reviewers. We strongly encourage code deposition in a community repository (e.g. GitHub). See the Nature Research guidelines for submitting code & software for further information.

Data

Policy information about availability of data

All manuscripts must include a data availability statement. This statement should provide the following information, where applicable:

- Accession codes, unique identifiers, or web links for publicly available datasets
- A list of figures that have associated raw data
- A description of any restrictions on data availability

All data are supplied in the supplementary information.

April 2020

Field-specific reporting

Please select the one below that is the best fit for your research. If you are not sure, read the appropriate sections before making your selection.

☒ Life sciences ☐ Behavioural & social sciences ☐ Ecological, evolutionary & environmental sciences

For a reference copy of the document with all sections, see nature.com/documents/nr-reporting-summary-flat.pdf

Life sciences study design

All studies must disclose on these points even when the disclosure is negative.

Sample size	This is an investigative and more qualitative study which elucidates the identity of molecular triggers. Pre-Covid parosmia was rather rare so 15 was rather arbitrary, and was dictated by the number of parosmics willing to travel to UoR. However it was deemed sufficient to prove our hypothesis that parosmia was triggered by odour-active aroma compounds. The same number of normosmics and post covid were recruited.
Data exclusions	For one pre-COVID-19 parosmic, the aetiology was post traumatic brain injury and they were excluded in the final data analysis
Replication	The olfactory tests are standard validated tests used routinely in clinics and needed no replication. GC-olfactometry was carried out twice to check the same compounds were detected a second time.
Randomization	There was no intervention, all groups were treated the same. Those recruited to the study prior to March 12th were deemed to have aetiologies other than Covid-19 (there is a 3 month lag before parosmia occurs) and those after May 2020 all reported sudden loss of smell and Covid-19 symptoms.
Blinding	No blinding was necessary as there was no intervention, all groups were treated the same.

Reporting for specific materials, systems and methods

We require information from authors about some types of materials, experimental systems and methods used in many studies. Here, indicate whether each material, system or method listed is relevant to your study. If you are not sure if a list item applies to your research, read the appropriate section before selecting a response.

Materials & experimental systems

n/a	Involved in the study
☒	☐ Antibodies
☒	☐ Eukaryotic cell lines
☒	☐ Palaeontology and archaeology
☒	☐ Animals and other organisms
☐	☒ Human research participants
☒	☐ Clinical data
☒	☐ Dual use research of concern

Methods

n/a	Involved in the study
☒	☐ ChIP-seq
☒	☐ Flow cytometry
☒	☐ MRI-based neuroimaging

Human research participants

Policy information about studies involving human research participants

Population characteristics	Pre-Covid, parosmia was a minor disease and we accepted all participants who fit the criteria. Age and gender are shown in Table 1
Recruitment	Participants were recruited through, facebook or personal contact. This may represent those more troubled by parosmia and may over-estimate the severity of parosmia.
Ethics oversight	University of Reading School of chemistry, food and pharmacy research ethics committee

Note that full information on the approval of the study protocol must also be provided in the manuscript.
