# Supplementary material for: Insights into the molecular triggers of parosmia based on gas chromatography olfactometry
Source: Commun Med (Lond). 2022 May 24;2:58. doi: 10.1038/s43856-022-00112-9 (PMC9130211; doi:10.1038/s43856-022-00112-9)
Supplement: Supplementary file 2 — Supplementary information [file 43856_2022_112_MOESM2_ESM.pdf]

Supplementary information for "Insights into the molecular triggers of parosmia based on gas chromatography olfactometry".- Parker, Kelly, Gane

Supplementary Methods

Materials. The following authentic aroma standards were purchased: **M3, T1** and **T2** from TCI (Oxford, UK); **P1**, **P2** and **P3** from Oxford Chemicals (Hartlepool, UK); **D1**, **D2**, **M1**, **M2**, **NT1, NT2** **P4**, **T4**, **X1**, **X2, X4**, and the C6-C25 alkane standard from Sigma (Poole, UK); **T3** in a capsule from Aroxa (Leatherhead, UK) and **T5** from Fluorochem (Hadfield, UK). Nescafé Original instant coffee sachets were purchased from Nisbets (Reading, UK). One box of instant Nescafé sachets (use by date August 2021) was purchased in September 2019 for use with the subjects between October 2019 and March 2020. A second box (use by date Oct 2022) was purchased in October 2020 to cross check the stability of the sachets over one year. Cocoa powder (Bournville, Cadbury, Bourneville, UK), skinless chicken breast fillet, smooth peanut butter (Tesco, Cheshunt, UK) and red bell peppers were purchased from a local supermarket.

Faecal samples were kindly prepared under Class 2 conditions by members of the food and microbial science group at the University of Reading. A faecal sample was collected from a healthy donor who had not consumed antibiotics within the previous six months. For transportation to the laboratory the sample was held under anaerobic conditions, using an Oxoid Anaerogen sachet (Oxoid, Hampshire, UK), for up to 2h before being frozen at -20 °C.

Extraction of coffee aroma. Fresh deionised water from a MilliQ system at 18.2 MΩ/cm resistivity was boiled in a kettle and 300 mL was added to the contents of the sachet (2.15 ± 0.05 g) in a 500 mL Duran bottle. The bottle was sealed, stirred for 2 min and an aliquot (3.0 ± 0.05 g) was transferred into an SPME vial. More concentrated extracts (contents of 1 sachet in 3 g boiling water) were also prepared for expert GC-O analysis and a detailed GC-MS analysis to aid identification of compounds. In both cases, the vial was equilibrated at 55 °C for 20 min and a preconditioned triple phase solid phase microextraction (SPME) fibre (50/30 µm divinylbenzene/carboxen on polydimethylsiloxane (Supelco, Poole, UK)) was exposed to the headspace at 55 °C for 20 min prior to analysis by GC-O.

Gas Chromatography-Olfactometry (GC-O). After extraction, the SPME device was inserted into the injection port of an HP7890 GC from Agilent Technologies (Santa Clara, CA, USA) coupled to a Series II ODO 2 GC-O system (SGE, Ringwood, Victoria, Australia). The SPME fibre was desorbed in a split/splitless injection port held at 280 °C. The columns employed were either an Agilent HP-5 MSUi capillary (30 m, 0.25 mm i.d., 1.0 µm df) non-polar column or a Stabilwax®-DA (30 m, 0.25 mm i.d., 0.25 µm df) polar column (Restek, Bellefonte, PA, USA). The temperature gradients were set as follows: 40 °C for 2 min, then a rise of 5 °C/min up to 200 °C and 15 °C/min from 200 °C to 300 °C (or 250 °C for the polar column), and the final temperature held for a further 19 min. Helium was used as carrier gas (2 mL/min). At the end of the column, the flow was split 1:1 between a flame ionisation detector (kept at 250 °C) and a sniffing port using 2 untreated silica-fused capillaries of the same dimensions (1 m, 0.32 mm i.d.). The flow to the odour-port was diluted with a moist make up gas.

Gas chromatography-mass spectrometry (GC-MS). An extract from a coffee prepared with one sachet in 3 mL of boiling water was extracted as above and analysed by GC-MS to aid identification of aroma compounds detected by GC-O and confirm their presence in the coffee extract. A7890A Gas Chromatograph coupled to a 5975C series GC/MSD from Agilent was used, equipped with either of the columns described above. The oven was held at 40 °C for 2 min, increased from 40 °C to 250 °C at a rate of 4 °C/min and then kept constant at 250 °C for 5 min. Helium was the carrier gas at a flow rate of 1.2 mL/min. Mass spectra were recorded in electron impact mode at an ionisation voltage of 70 eV and source temperature of 220 °C. A scan range of m/z 20-300 with a scan time of 0.69 s was employed and the data were controlled and stored by the ChemStation software (Agilent, Santa Clara, CA).

Identification of odour-active compounds. Linear retention indices were calculated by comparison with the retention times of C_6-C_{25} n-alkane series analysed on the same day using the same conditions as for sample analyses (Supplementary Table 1). Aromas eluting from the GC-O were identified by comparing their LRIs, mass spectra and the odour as described by the experts with those of authentic compounds on two columns of different polarity. Mass spectral libraries, such as NIST 2011 and

Inramass (INRA, France), were used for primary identification of compounds in the coffee extract using ChemStation software (Agilent, Santa Clara, CA). In most cases, authentic compounds were analysed using the same chromatographic method to confirm their identity by comparison of their mass spectra, LRI, and odour quality. Identification was confirmed by GC-MS on a Stabilwax column. For compounds at concentrations below the detection limit of the GC-MS, odour character and LRI were used.

Additional samples. All additional samples were prepared as for coffee with the following modifications. Cocoa: 3g of cocoa powder was dissolved in 10 g boiling water, stirred and a 3 g aliquot was used for extraction. Meat: a lean breast fillet, thickness 1 cm was grilled for 3 min on either side using a Cuisinart grill (Stamford, CT) set on high. Finely chopped meat (3 g) was used for extraction. A 50:50 slurry of peanut butter (3 g) was used for extraction. Finely diced red pepper (3 g) was extracted at 40 °C prior to desorption. The faecal sample was thawed, mixed with an equal weight of water, and 3 g transferred to an SPME vial. Chromatography conditions for all samples remained the same as for coffee.

Statistics. The age of the pre-COVID-19 parosmic participants, post-COVID-19 parosmic participants and non-parosmic participants was analysed using Kruskal-Wallis followed by pairwise comparison using Steel-Dwas-Critchlow-Fligner (significance set at 0.05) to determine significant differences between the groups, whereas Anova followed by Tukey HSD ($p=0.05$) was used for TDI scores. Principal component analysis was carried out on intensity data. All statistical analyses were carried out using XLSTAT version 20201.1.1 statistical and data analysis solution (Addinsoft 2020).

Supplementary Figure 1 Flavour wheel provided to all participants during GC-O session

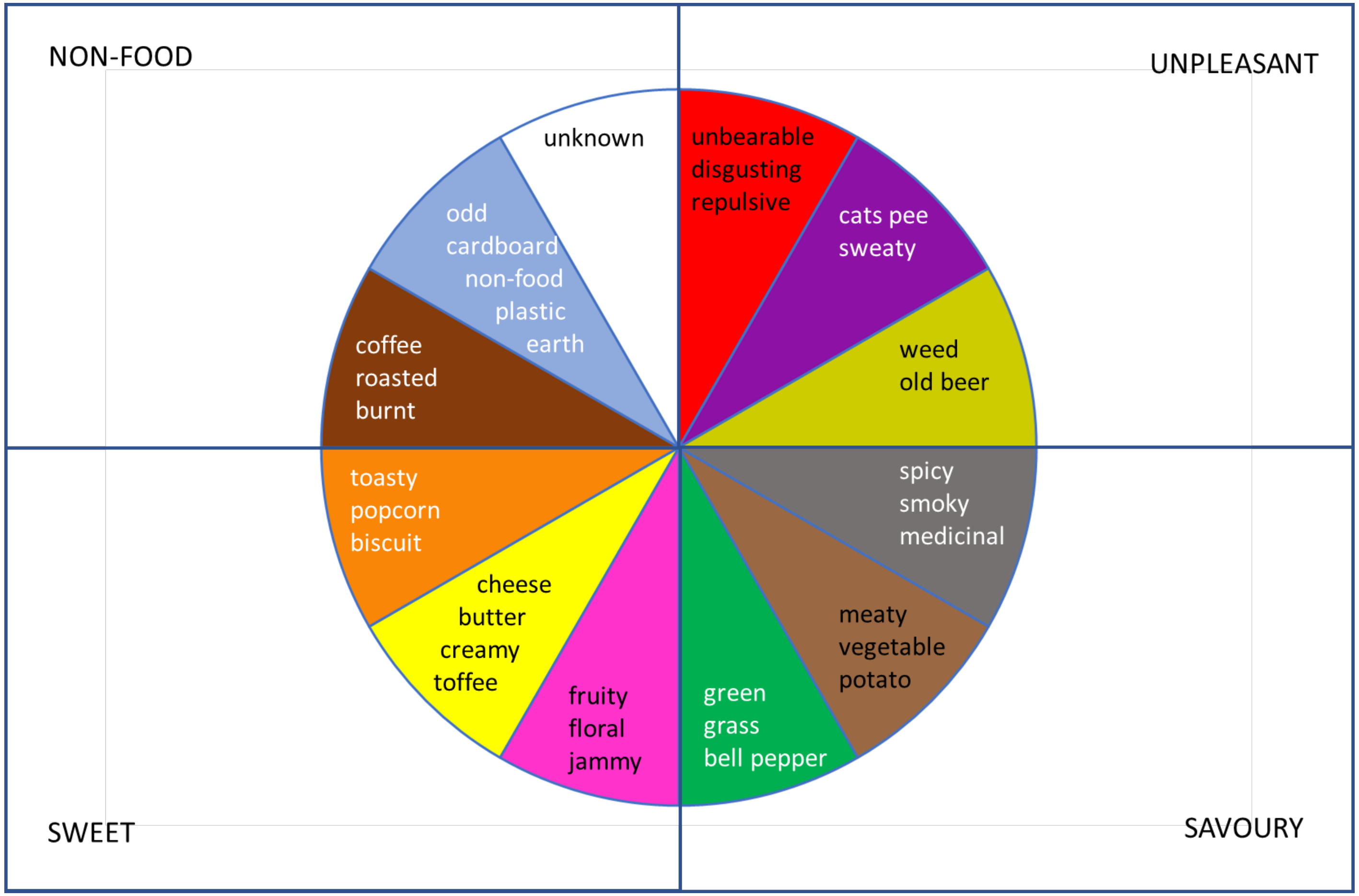

Supplementary Table 1 Intensity Data for Fig. 3

		Intensity scores for Pre-COVID-19 participants (gLMS[a] scale)												Intensity scores for Post-COVID-19 participants (gLMS scale)													
**Compound**	PPT	1	2	3	4	5	6	7	8	9	10	11	12	1	2	3	4	5	6	7	8	9	10	11	12	13	14
	Code																										
2-furanmethanethiol	**T1**	44	32	13	39	41	47	38	62	15	7	8	0	17	27	17	17	89	33	44	32	9	35	0	3	11	6
2-ethyl-3,6-dimethylpyrazine	**P1**	38	0	26	13	42	2	0	0	25	0	9	0	17	8	0	16	35	0	19	0	4	17	35	32	2	6
2,3-diethyl-5-methylpyrazine	**P2**	51	0	28	0	18	26	51	0	19	1	39	34	35	8	0	17	25	44	0	17	15	6	28	11	0	17
2-furanmethyl methyl disulfide	**D1**	39	22	0	10	33	15	3	37	2	3	0	1	17	10	6	6	8.9	0	6	17	9	0	0	0	0	0
2-methyl-3-furanthiol	**T2**	21	35	9	1	44	7	25	43	2	3	8	0	17	13	17	11	100	33	0	0	0	35	0	0	0	4
2-methyl-3-furyl methyl disulfide	**D2**	32	22	8	13	42	22	39	42	6	0	0	38	17	6	17	8	35	0	0	17	0	6	35	0	0	0
2-ethyl-3,5-dimethylpyrazine	**P3**	42	0	46	42	44	0	0	59	0	0	39	38	35	14	0	6	0	53	27	6	20	18	0	0	2	17
3-methyl-2-butene-1-thiol	**T3**	26	28	9	31	26	32	34	39	4	2	0	0	17	30	17	6	89	4	8	44	16	0	8	0	0	2
2-ethyl-3-methoxypyrazine	**M1**	40	0	26	23	0	54	15	0	25	0	0	0	17	8	0	0	5.7	0	0	0	4	0	0	8	0	2
2-isobutyl-3-methoxypyrazine	**M2**	32	22	22	0	32	17	0	43	7	2	8	22	6	8	35	0	17	0	0	6	4	0	0	0	0	6
3-mercapto-3-methylbutan-1-ol	**T4**	21	15	15	39	0	19	27	72	8	0	0	0	17	8	0	0	35	25	0	0	0	0	0	0	0	6
sotolone	**X1**	51	0	32	26	24	20	0	0	0	0	8	0	0	8	0	11	0	44	17	0	0	0	0	0	0	0
3-mercapto-3-methylbutyl formate	**T5**	31	11	43	33	44	66	34	64	19	7	9	0	0	9	0	2	0	51	0	17	0	0	0	0	0	0
guaiacol	**X2**	40	8	48	24	0	73	0	0	68	4	0	37	17	0	0	0	35	44	33	40	0	6	0	0	0	0
trimethylpyrazine	**P4**	0	22	40	20	0	48	0	0	0	0	3	0	17	8	0	0	22	0	6	0	0	0	0	0	0	6
unknown LRI 981	**X3**	25	28	34	13	43	51	0	44	6	13	0	0	0	8	0	11	0	24	0	0	0	0	0	0	0	0
2-isopropyl-3-methoxyprazine	**M3**	0	22	41	21	44	76	15	44	0	1	0	0	17	9	0	6	47	41	0	11	9	0	0	0	0	0
2,3-butanedione	**X4**	18	20	8	7	0	32	25	23	7	3	7	0	2	0	6	0	0	6	6	0	0	0	0	0	0	2
4-ethylguaiacol	**NT1**	8	13	8	11	41	0	0	62	0	2	0	0	17	3	35	0	0	0	0	0	0	0	0	0	0	0
(E)-β-damascenone	**NT2**	13	21	16	1	27	0	4	0	0	0	0	0	6	0	6	0	35	0	0	0	0	0	0	0	0	0
Olfactory function	**OF**	36	35	29	31	26	26	30	35	31	26	13	32	37	36	30	38	27	27	29	25	19	32	24	23	15	28

[a]general labelled magnitude scale

Supplementary Note: Pre-screening Questionnaire Part 1

What is your title?
What is your full name?
What is your full address (including postcode)?
What is your date of birth?
Please indicate your ethnicity
What is your height (please indicate units)?
What is your weight (please indicate units)?
How would you describe your gender?
What is your daytime telephone number?
What is your evening telephone number?
What is the best time to call?
Are you a current smoker?
What is your email address?
Do you use emails on a regular basis?
How did you hear about the study?
As far as you know, do you have chronic sinusitis?
As far as you know, do you have olfactory dysfunction?
Have you had surgery to your nose or head?
Please list any current medication you're taking. If no medication is taken, please write 'none'.
Are you prepared to spend a day at the Flavour lab at University of Reading?
Do you experience a problem with your sense of smell or taste?

<u>Pre-screening Questionnaire Part 2 for those reporting smell disorders</u>
Does your problem with your sense of smell or taste relate to your (select one answer)
- Sense of Smell
- Sense of Taste
- Both smell and taste

Can you tell the difference between salt and sugar? Yes/No
When did you problem start? (select one answer)
- Less than 3 months ago
- 3-12 months ago
- 1-2 years ago
- More than 2 years ago

How did the problem start? (select one answer)
- Slowly
- Suddenly
- I don’t know

How has your problem changed since it started? (select one answer)
- There’s been an improvement
- No change
- It’s got worse
- Not sure

What do you think might have caused it? (select one or more answers)
- Accident
- Nasal polyps
- Cold or infection
- Surgery
- I was born without a sense of smell
- Other
- I don’t know

Do you have any of these signs/symptoms? (select one or more answers)
- Stuffy nose
- Allergies
- Sneezing
- None of these
