# Supplementary material for: Insights into the molecular triggers of parosmia based on gas chromatography olfactometry
Source: Commun Med (Lond). 2022 May 24;2:58. doi: 10.1038/s43856-022-00112-9 (PMC9130211; doi:10.1038/s43856-022-00112-9)
Supplement: Supplementary file 5 — Description of Additional Supplementary Files [file 43856_2022_112_MOESM5_ESM.pdf]

Description of Additional Supplementary Files

File Name: Supplementary Data 1

Description: Confirmation of Identity of Molecular Triggers of Parosmia

File Name: Supplementary Data 2

Description: Participant Data: Demographics, answers to pre-screening questionnaire, Sniffin'Stick Scores and GC-O data
